# Nutrient Density and Microbial Safety of Open-Air-Dried Beef Meat and Its Biochemical and Organ Histopathology Effects in Albino Rats: A Promising Ingredient for Complementary Food Formulation

**DOI:** 10.1155/2023/2202312

**Published:** 2023-02-21

**Authors:** Kifle Habte, Meseret Azene, Yohannes Chanyalew, Samson Girma, Chala Bashea, Asrat Yehualshet, Getamesay Behailu, Abiy Abebe, Masresha Tessema

**Affiliations:** ^1^Department of Food Science and Nutrition Research Directorate, Ethiopian Public Health Institute, Ethiopia; ^2^Center for Food Science and Nutrition, Addis Ababa University, Ethiopia; ^3^Thematic Director of Health and Nutrition, Save the Children International, Country Office, Ethiopia; ^4^Clinical Chemistry Reference Laboratory, Ethiopian Public Health Institute, Ethiopia; ^5^Traditional and Modern Medicine Research Directorate, Ethiopian Public Health Institute, Ethiopia

## Abstract

**Introduction:**

Dried beef meat is a major source of essential fatty acids, minerals, and vitamins that are digestible and absorbable, thus could be a potential source of nutrients in complementary food formulations. Composition, microbial safety, and organ function tests were analyzed, and histopathological effect of air-dried beef meat powder was determined in rat model.

**Methods:**

Three groups of diets were given for the three groups of animals: (1) standard rat diet, (2) meat powder+standard rat diet (1 : 1 formulation), and (3) dried meat powder. A total of 36 Wistar albino rats (18 males and 18 females) of 4-8 weeks old were used and randomly assigned to the experiments. After acclimatization for one week, the experimental rats were followed for 30 days. Microbial analysis, nutrient composition, organ histopathology (liver and kidney), and organ function tests were conducted from serum samples taken from the animals.

**Results:**

Protein, fat, fiber, ash, utilizable carbohydrate, and energy contents of meat powder on a dry weight basis were 76.12 ± 3.68, 8.19 ± 2.01, 0.56 ± 0.38, 6.45 ± 1.21, 2.79 ± 0.38 g/100 g, and 389.30 ± 3.25 kcal/100 g, respectively. Meat powder could be also a potential source of minerals such as potassium (766.16 ± 77.26 mg/100 g), phosphorus (150.35 ± 16.26 mg/100 g), calcium (18.15 ± 7.80 mg/100 g), zinc (3.82 ± 0.10 mg/100 g), and sodium (123.76 ± 32.71 mg/100 g). Food intakes were lower in MP group compared to the others. According to organ histopathology results, animals fed with the diet have shown normal values, except rise in alkaline phosphatase (ALP) and creatine kinase (CK) in groups fed with meat powder. The results of organ function tests were all within the acceptable ranges and comparable with their counterpart control groups. However, some of the microbial contents of the meat powder were not within the recommended level.

**Conclusion:**

Dried meat powder has a higher amount of nutrients, which would be a potential recipe in complementary food preparation that can support to reduce child malnutrition. However, further studies need to be conducted on the sensory acceptability of formulated complementary foods containing dried meat powder; also, clinical trials are aimed at observing the effect of dried meat powder on child linear growth.

## 1. Introduction

Undernutrition is a major cause of morbidity and mortality among under-five children [1]. Globally, about 180 million under-five children are stunted, in which nine out of ten are living in developing countries such as Africa and Asia [2]. It affected about 33% of under-five children residing in sub-Saharan countries [3]. According to the Ethiopian Mini Demographic and Health Survey (EDHS) in 2019, the prevalence of stunting, underweight, and wasting in under-five children were 37%, 21%, and 7%, respectively [4], whereas EPHI [5] indicated that the prevalence of anemia, iron deficiency anemia (IDA), and zinc deficiency in under-five children was 34.4%, 12.3%, and 35%, respectively, and extensively studied that micronutrient deficiency in children is mainly caused either by inadequate diet diversity and nutrient intake and infections or both. The consequence could be detrimental, ranging from poor physical and mental development to low school performance [6, 7] and reduced productivity and low economic growth later in life [8]. One of the approaches to curb malnutrition is diversifying diets with animal source foods [9], since animal source foods are the principal source of densely and readily bioavailable nutrients for daily requirements, particularly for growing children [10]. But according to the Ethiopian national food consumption survey, the intake of animal source foods in Ethiopia was very marginal, especially in children [11]. Beef meat is one of the largely consumed food globally, which is rich in protein, iron, selenium, zinc, copper, manganese, sodium, vitamin A, vitamin B complexes, and folic acid [8, 12]. In addition, the high-quality proteins, essential amino acids, micronutrients, and polyunsaturated fatty acids (such as 18% in omega-6 and 17% in omega-3) found in meats have key contribution to health, digestibility, and metabolic benefits [12, 13]. However, despite being loaded with nutrients, beef meat is among the highly perishable food by microbial contamination due to high water activity, only slightly acidic in pH, high glycogen, and protein content [14–16]. These properties give a red meat shorter shelf life [14], thus need to be carefully produced by adhering with good manufacturing (GMP) and hygienic practice (GHP) throughout the value chain [17], maintaining the food storage and preservation techniques to extend the shelf life [15].

Drying meat is one of the most common methods globally used to extend the shelf life as it reduces moisture and water activity easily [18]. The process also helps to ease packaging, reduce transportation and storage costs [14], and also facilitate to make meat in a powder form, thus to sprinkle it in a routine complementary foods of children [19]. In Ethiopia, dried meat locally known as “Quanta” slices of long strip meat is dried in the open air inside a house by hanging over ropes; rarely table salt or pepper powder is spread on the slices while hanging. The drying process varies and usually takes an average of 10 days [20]. In Ethiopia, feeding meat to young children particularly those who are 6-18 months is restricted mostly due to a traditional belief that the food is very heavy for a child's intestine and might also cause choking; however, rarely, few communities have been used in powder form to add it in child's porridge [21].

It could be a motivating experience for a child to use meat powder in complementary food preparation which could support healthy and sufficient child growth and eradicate malnutrition, particularly in area where malnutrition is the most prevalent and low intake of animal source foods. However, meat powder production, preparation, and storage process could be amenable for microbial proliferation and trace metal contamination (such as arsenic, cadmium, and lead); aflatoxin, dioxin, nitrites, botulinum toxin, biogenic amines, and organochlorine pesticides might be the contaminants linked with the product [22–25]. Therefore, it requires to stick with food safety principles in critical place of the value chains to prevent diarrhea, food poisoning, and toxic substance exposure. The present study estimated the nutritional composition and selected microbial safety parameters in meat powder and meat powder mixed diets in comparison with standard diet. The study also examined the amount of food intake, change in weight, and toxicity of dried meat powder after routine subchronic exposure through animal organ histopathology, organ function tests, and observation of clinical symptoms.

## 2. Materials and Methods

### 2.1. Sources of Meat and Preparation

Raw red beef meat was purchased from Burayu city slaughter and distributor house, Oromia region, Ethiopia. The purchased meat was sourced from brisket, chuck, round, sirloin, and plate parts of the animal, and composite samples were taken after removing the external fat, ligaments, and bone from the red beef meat and sliced into pieces by a research technician. Fresh beef meat samples were purchased in three different weeks from January to February 2019, to avoid long time storage of the meat. Purchased samples were stored in a sterile ziplock plastic bag, put in an icebox, and transported to Ethiopian Public Health Institute (EPHI) for laboratory analysis. Meanwhile, the samples were dried by hanging them on a string of ropes inside a clean kitchen with circulating fresh air. Microbial analyses were conducted for standard rat diet (group I), meat powder mixed with a standard diet (50% each) (group II), and meat powder (group III), whereas nutritional analysis was conducted only for dried meat powder.

### 2.2. Meat Drying

Red meat slices were placed on large platters, then hanging up in a thread till drying (~10 days) in the open air at room temperature (21-23°C) similar to Ethiopian traditional dry meat (quanta) preparation as described in [21]. Finally, dried meat samples were grounded into powder form using animal feed pellet machine, of capacity 20-35 kg/hour for pelleting. At the beginning, metal pounding (mortar and pestle) was used to support course milling, and then, a small electric grinder (700/800 T) with 3600 rpm and 30-300 mesh was used for milling. The powder was put in a glass bottle and stored in a -20°C fridge until microbial and nutrient analyses.

### 2.3. Experimental Animals and the Diets

**Figure 1:**
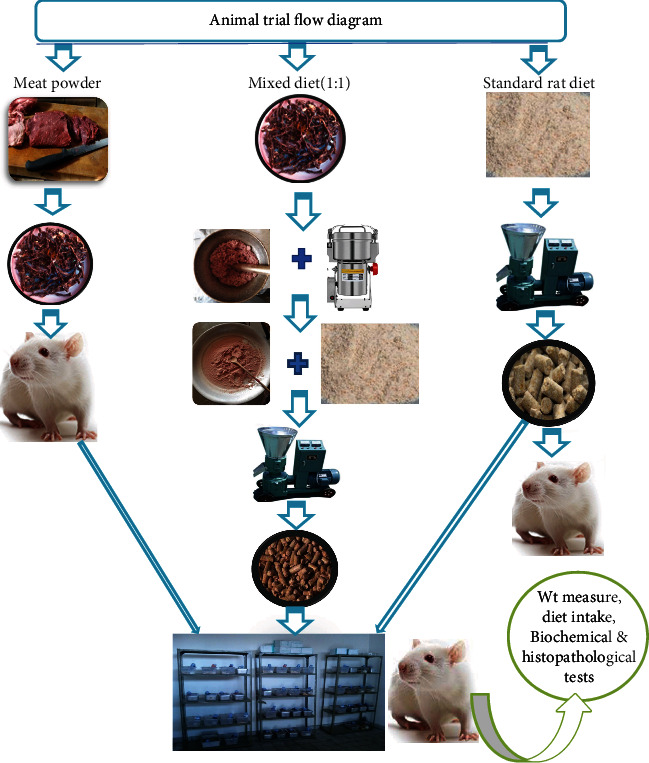
Schematic diagram of feed preparation and animal trial setup.

Wistar albino rats of both sex 4-8 weeks old were obtained from the vaccines and diagnostic production department of EPHI. However, before conducting the study, ethical clearance was requested, and the study protocol ethical approval was obtained from EPHI institutional review board (IRB) with ethical approval number (EPHI-IRB-111-2018). The animals were housed in individual plastic cages in a 12-hour light-to-dark cycle at room temperature (21-23°C). The trial was performed according to the guidelines for laboratory animal handling and care [24]. The experiment was conducted according to the general guidelines for designing and conducting toxicity studies [26, 27] and short-term toxicity studies with rodents [26]. The flow diagram of the animal trial setup is shown in Figure 1. All animals were acclimatized to the laboratory setup for a week before starting the actual trial. Rats were divided into three groups (groups I, II, and III), each group composed of 12 individual albino rats with an equivalent ratio of male (six) and female (six) distribution to avoid sex-related cofounding; according to a recommendation in short-term toxicity studies with rodents by FDA [26], the number of experimental animals can be reduced to 10 when long-term studies are anticipated, but to avoid any chance of rat missing or withdrawal in between due to mortality, 2 rats were added as backups. Group I rats were those who were fed a standard diet (SAFE A04, France) daily, given 20 g/day. Group II rats were those fed mixed diet (10 g red meat powder+10 g standard diet), in a pellet form given 20 g/day. Group III rats were those fed dried red meat only, given 20 g/day. As the dried beef meat powder alone was difficult to make a common rat diet shape (pellet form), those assigned to the meat powder only were given the red dried meat directly without making its powder form. To make it suitable for lab analysis, the dried meat was milled to powder form; it also was possible to mix the meat powder with the standard rat diet (1 : 1) ratio; the mixture of pellets (a densely cylindrical intact, ~10 mm diameter and 20 mm length) was prepared using an animal feed miller machine.

The animal trial lasted for 30 days. Three different diets were taken as an independent variable, aimed to associate against continuous dependent variable (biochemical and histopathological results). Descriptive statistics using SPSS and one-way ANOVA was performed for inferential statistical analysis. The nutrient composition of the standard rat diet is indicated in Table 1.

### 2.4. Rat Body Weight and Diet Intake

Animals were followed daily whether they show any unique clinical symptoms. Amount of food intake was measured daily; leftover foods were collected and deducted from the daily ration. The cages were cleaned from feces in 24 hours. Water was given as *ad libitum* for all animals in the trial period. Animals were weighed initially and at the end of the 30-day feeding period. The percentage change in weight was calculated according to

(1) $$\begin{array}{c} \text{Body weight change }\left(\%\right)=\frac{\text{Final rat weight}-\text{Initial rat weight}}{\text{Initial weight }}. \end{array}$$

Food efficiency ratio (FER) was determined according to

(2) $$\begin{array}{c} \text{FER}=\frac{\text{Total weight gained }\left(\text{g}\right)}{\text{Total diet intake }\left(\text{g}/\text{day}\right)}, \end{array}$$

where FER is the food efficiency ratio.

### 2.5. Biochemical Analysis in Serum Samples

After 30 days of the feeding period, rats were fasted for 24 hours and anesthetized with diethyl ether; blood samples were collected by cardiac puncture. The blood samples were centrifuged at 3000 rpm for 10 min; after being separated from the whole blood, the serum was stored at a temperature of -80°C until clinical chemistry analysis was run. Liver function tests such as aspartate transaminase (AST), alanine transaminase, alkaline phosphatase (ALP), albumin, and bilirubin total (BILT); enzyme tests such as *α*-amylase (AMYL) and lipase [28]; and heart function tests such as C-reactive protein (CRP), highly sensitive (CRP-HS), troponin, and creatine kinase (CK) were conducted. In addition, kidney function tests like urea and creatine were examined according to analysis parameters indicated in [1, 28, 29]. All the biochemical tests were analyzed using Cobas® 6000 analyzer series (Roche Diagnostics GmbH).

### 2.6. Organ Histopathology Examination

Biopsy (portions) of the liver and kidney tissues from each rat was taken cautiously. After adding 10% formalin, the tissues in paraffin are fixed and stained with hematoxylin and eosin (H&E) dye in the EPHI pathology laboratory. The control and treatment animal organ slides were subjected for examination through the binocular light microscope (Olympus CX41, Japan) at a magnification of 20x and 40x. The micrographs of organ histopathology were analyzed and interpreted by a pathologist according to similar method used in [30].

### 2.7. Proximate Analysis

The proximate composition (moisture, protein, fat, energy, ash, and dietary fiber) content of red meat powder was determined according to the Association of Official Analytical Chemists [31] as the method described [31]. Moisture content was conducted using oven drying; reading in analytical balance was taken after stable reading shown in the balance (AOAC 930.15, 2016). Ash content was determined using a muffle furnace at a temperature of 550°C for 8 hours and measured after white ash produced according to AOAC 923.03 (2016). The protein content was analyzed by the Kjeldahl method. Nitrogen to protein content was converted using 6.25 conversion factor, since total nitrogen data is obtained from the analysis. Fat content was determined gravimetrically using petroleum ether extract in a Soxhlet apparatus according to AOAC 2003.06 (2016). Dietary fiber content was determined gravimetrically by dissolving the sample with sulfuric acid followed by sodium hydroxide (ES ISO 5498 : 2002) [32]. Total carbohydrate content was obtained by the difference method. The total energy was calculated by multiplying the average value of both crude protein and total carbohydrate content by 4, but crude fat by 9.

### 2.8. Mineral Composition

Mineral contents of dried red meats were determined following the method of AOAC, 999.11 [33], after ashing of the dried samples in furnace at 550°C. Calcium, iron, and zinc were determined by using Microwave Plasma Atomic Emission Spectroscopy (MP AES, Agilent-4200) after digestion and cooled, and the samples were filtered. The sodium and potassium were determined using flame photometer (JANEWAY, PFP 7, Essex UK). This was done by adding 5 mL of 6 M HCl followed by 15 mL of 3 M HCl in the sample, gentle heating; then, the solution was allowed to boil. The samples were digested, cooled, and filtered, after being added with 5 mL of lanthanum chloride solution. Determination of calcium, iron, and zinc content was done at an absorbance of 393.4, 372, and 213.9 nm, respectively. Total phosphorus content was determined using UV-VIS spectroscopy (Shimadzu, UV-1800) by a molybdovanadate method according to the procedures used in AOAC 995.11 : 2016 at a wavelength of 823 nm [34].

### 2.9. Microbial Analysis

A 25 g of dried beef meat powder was mixed with 225 mL of buffered peptone water (Oxoid, UK) with a nonselective enrichment followed by serial dilutions using 9 mL of 1% buffered peptone water and normal saline water. Total coliform and fecal coliform counts were conducted using pour plate method, whereby 1 mL of each serial dilution was pipetted into a Petri dish and then mixed with a 5 mL of tryptone soya agar; the agar with 10-15 mL of violet-red bile lactose agar (VRBLA) (Oxoid, UK) was covered. For the total coliform count, the plates were incubated at a temperature of 37.0°C and for fecal coliform incubated at 44.0°C for the duration of 24 ± 2 hours. For the presumptive coliform and fecal coliform, approximately 1 mm diameter size colonies with pink color were counted. Confirmatory tests were done by taking five representative colonies and inoculating to brilliant green bile broth and Escherichia coli (E. coli) broth, respectively, for coliform and fecal coliform count. E. coli count was conducted after fecal coliform count was made using E. coli broth and nutrient broth according to [35].

Aerobic mesophilic count was determined using a pour plate method, kept at 30°C for 48 hours. Sample of buffered peptone water (BPW); then, serial dilutions were made; plates were put at 30°C for 72 hours [36]. Colonies were counted in each of the Petri dishes with a count between 30 and 300 colony-forming units (cfu). Salmonella was detected according to the method described by [37]. Samples were nonselectively enriched in buffered peptone water (BPW). After 24-hour growth at 36±1^0^c, selectively enriched using selenite cysteine broth and rappaport vassiliadis enrichment broths, then incubated at 35±1^0^c. After purification and biochemical identification, qualitative results were presented as present and absent. Mold and yeast counts were performed by spread plate techniques; 0.1 mL of the respective diluted sample was added to prepare rose bengal agar supplemented with chloramphenicol. Samples were spread using a spreader after being incubated at 22°C for 5-7 days according to the method described for total mold and yeast counts (cfu/g) [38]. Microbial load of MP was checked after completing the process of slicing, air-drying, and making the dried meat into powder form. The same process was followed for a standard diet, except slicing which does not need standard feed. Final microbial analysis was done to estimate the degree of microbial contamination at the final product after many traditional meat powder preparation processes as similar steps as food preparation at household level aimed to sprinkle the MP in complementary foods of children in the future.

## 3. Results

### 3.1. Body Weight, Food Intake, and Food Efficiency Ratio of Rats

Experimental animals were given three types of diets independently for the trial period (1 = standard rat diets, 2 = mixed diets (1 : 1) ratio, and 3 = red dried beef meat). The rats were put in independent cages, and the leftover food samples were removed and deducted from the daily ration. Food intake, weight gain, and food efficiency ratio of rats fed with the three types of diets are presented in Table 2. A one-way ANOVA analysis showed that the food intake and food efficiency ratio of rats among the three groups were significantly different (*p* < 0.05), but the weight gain/weight change (g) was not significant (*p* > 0.05). Percent weight change was the highest in mixed diets (98%), but lowest in group I (61%). Daily average food intake in standard rat diets, mixed diets, and dried meat was 18.37 g, 17.57 g, and 13.28 g, respectively. The average food intake in standard rat diets and mixed diets was not statistically significant compared to dried beef meat. Conversely, the food efficiency ratio was significantly different among the groups (Table 2).

### 3.2. Diet Influence on Histopathology of the Organs

**Figure 2:**
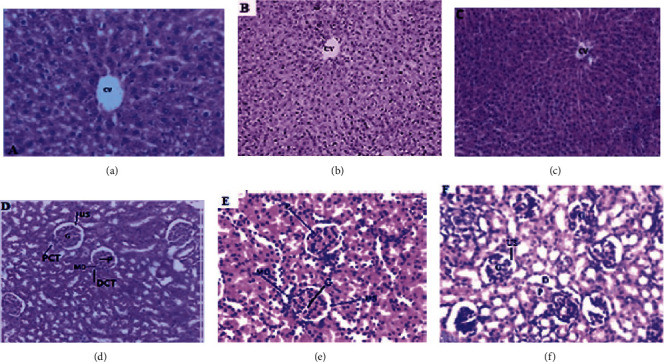
Liver and kidney histopathology of the rats. The upper three (a–c) are showing the photomicrographs of liver of standard diet/control, mixed, and meat powder, given 20 g of each types of the diets per day, respectively. CV: central vein. Kidney histopathology of rats that consumed the three diets (d–f) in the same order as above. The tissues were stained in H and E, examined in a microscope with power of 400x total magnification. PCT: proximal convoluted tubule; DCT: distal convoluted tubule; US: urinary space; G: glomerulus; MD: macula densa; P: podocytes.

After the trial period, rats were examined for kidney and liver histopathology. The three types of diets consumed by the rats demonstrated absence of structural alterations in the organs. The microstructures of the portal triad, the sinusoids, the hepatocytes, and the bile duct system in the liver looked quite normal (Figure 2). Similarly, no histopathology effect was examined in all rats and the groups. The glomeruli features were normal in the kidney. No capillary loop thrombi, absence of necrosis, or crescents were observed. In addition, no significant organ pathology identified nor were tubules and interstitium was examined. There was an absence of pathology in the vessels showing normal reticulin stains. The renal cortex, efferent arteries, glomerular capillaries, and Bowman capsule have not shown any structural deformations with normal nephrogenic zone, and the medulla in all blocks were examined. Figures 2(a)–2(c) show the photomicrographs of the liver, and Figures 2(d)–2(f) show the photomicrographs of the kidney.

### 3.3. Effects of Diets in Biochemical Status

Fasting blood samples (serum) of the animal were analyzed. The results are presented in Table 3.

Analysis data of AST, ALB, AMYL, LIP, CRP-HS, troponin, and creatinine data were not significantly different (*p* > 0.05) in the three groups of experiments. Liver and kidney test results like ALT, AST, ALB, urea, and creatine agreed with the previous study [39, 40]. Troponin values were within the reference ranges according to the normal range shown in [39].

The normal range of ALP is 44 to 147 U/L [41]. But ALP level in MP-fed rats was borderline high and comparably higher than the other two groups, which might be due to the accumulation of fats in the liver because of the consumption of only dry meat (the fat content is too much higher than the standard diet; see Tables 1 and 4) for thirty days [41]. Studies explained that serum CK tends to increase in case of liver disease or pathological liver tissue [42–44]. According to Meffert et al. [43], the median CK of male and female Wistar rats is 435 and 270 U/L, but with a maximum of 829 and 828 U/L, respectively. During muscle injury, CK levels will rise and may correlate with the risk of AKI. However, the risk of developing AKI is usually low when the CK level is below 10,000 U/L [45]. In the present study, relative higher CK level was observed in MP consumers, in which monotonous way of meat consumption might lead to muscle and bone weakness due to its relative lower level of calcium and phosphorus (40 and 4 times lesser, respectively) than the standard diet (see Tables 1 and 4).

One-way ANOVA result indicated that compared to those who consumed standard diet and mixed diets, meat powder consumers exhibited significantly higher ALT, ALP, CK, and urea serum levels at *p* < 0.05. Mean difference on Tukey's test of the liver biomarkers indicated that ALT, ALP, and BILT (bilirubin total) in standard diet and mixed diet were not significantly different (*p* < 0.05).

For all experimental rats including those who consumed standard diets, no biochemical results exceeded the acceptable ranges, except ALP and CK shown in dry meat. Excess urea results in those animals that consumed meat powder indicate that extra meat and protein consumption might rose the excretion compared to the other two diet groups.

### 3.4. Microbiological Analysis

The microbial data analysis of the three groups of diets (standard rat diet, mixed diet, and dry meat powder) is shown in Table 5. The overall mean of aerobic mesophilic count in standard diet, mixed diet, and dry meat powder was 4.74 ± 0.31, 5.53 ± 0.46, and 7.85 ± 0.12 log (cfu/g), respectively. Total coliform count in the diets was 3.14 ± 0.38, 4.11 ± 0.31, and 6.47 ± 0.61 log (cfu/g), respectively. Fecal coliform count was detected in final weeks of the standard diets, but in all of the mixed diet with mean of (3.46 ± 0.04) and in dry meat powder (6.2 ± 0.33) log (cfu/g). E-coli was not present in the first and second weeks of the standard diet, at first weeks in dry meat powder, and at the second week in mixed diet. The average total mold counts in standard, mixed, and meat powder diets were 2.71 ± 0.38, 2.64 ± 0.48, and 2.25 ± 0.71 log (cfu/g), respectively. Yeasts and Salmonella were not detected in all three types of diets.

### 3.5. Meat Powder Nutrient Analysis

Nutrient compositions of dry red meat powder from samples collected in different weeks are shown in Table 4.

The average result of macronutrient and micronutrient contents of meat powders (g/100 g, dry weight basis) was 76.12 ± 3.68 (crude protein), 8.19 ± 2.01 (crude fat), 6.45 ± 1.21 (total ash), 8.21 ± 0.54 (moisture), utilizable carbohydrate (2.79 ± 0.38), and 389.30 ± 3.25 (energy) (kcal/100 g). In addition, the study showed that dry meat powder is composed of various micronutrients such as potassium (766.16 ± 77.26), phosphorus (150.35 ± 16.26), calcium (18.15 ± 7.80), zinc (3.82 ± 0.10), iron (2.80 ± 0.09), and sodium (123.76 ± 32.71 mg/100 g). Mean proximate composition of dry meat powder (duplicate samples of each analyzed at different weeks) is presented. This nutrient information is helpful to estimate the amount of powder to be added, and the daily nutrient can be fulfilled when feeding children (6-23 months) in their routine complementary foods. The nutrient requirement in the daily ration was taken from [46] in the middle value (50 g of daily ration for 6-23-month-old children). Accordingly, if a child takes a tablespoon (~14 g) of meat powder three times a day (breakfast, lunch, and dinner), thus the daily nutrient requirements and the amount can be achieved as shown in Table 6.

## 4. Discussion

Experimental rats consumed the diets efficiently, but relatively lower daily intake was recorded in group III (dry meat-consuming rats) in the study period. There was rapid weight gain in all groups of rats compared to their baseline weight, but the increment was not statistically significant among groups. The present result shows that meat powder is not only a source of optimum level of macronutrients such as protein, fat, and energy but also promising for micronutrients like phosphorus, potassium, and zinc. Consuming “dried beef meat alone” and “mixed with standard rat diet” had not affected the histopathology (liver and kidneys) of the rats nor affected the organ function tests compared to the control groups. Those children fulfilling adequate dietary diversity in Ethiopia is very marginal (8.6%) [39]; the same is true for daily consumption of animal source food, particularly meat only (6%) in under 2-year-old children, which is one of the main factors for substandard dietary diversity [47]. This is often mentioned as one of the major cause of child stunting in Ethiopia [48].

However, the majority of mothers/caregivers restrict meat for the children (6-23 months) complementary foods as culturally they believe that the children are unable to swallow and digest in their intestine [49, 50]. However, sprinkling a specific amount of (2-3 tablespoon) of meat powder in complementary food could not only rise the dietary diversity of children but also could play a vital role in the prevention and management of child malnutrition (stunting, wasting, and micronutrient deficiency) [51]. Nonetheless, beginning from raw meat production up to meat powder preparation and consumption may be liable to microbial contamination, unless the value chains are kept hygienic or prepared in an advanced way of production to protect proliferation of pathogenic microorganisms [52]. According to food safety authority guideline, the satisfactory level threshold of total bacterial count in raw beef meat should be <105 cfu/g and the acceptable range is 105-106 cfu/g. For E. coli count, <20 cfu/g is satisfactory, whereas between 20 and 100 cfu/g is acceptable. However, for Salmonella, it should be absent in 25 g of the sample (zero tolerance) [38].

Some of the microbial load of the present study surpassed the tolerable ranges, for example, total viable count (>106 cfu/g) [53] and E. coli (above the acceptable range), but yeasts and Salmonella were absent in 25 g sample. During preparation of the samples, tap water was used in making the rat diets into cylindrical pellet for the rat, but among others, the water microbial level was not analyzed and might be one of the sources for cross contamination, as aerobic mesophilic bacteria and total coliform were identified in the standard diets while pasting it with tap water which is supposed to be absent. The microbial load of raw beef meat collected from different parts of Tigray region, in Ethiopia, reported that total aerobic mesophilic count was between 5.4 and 8.2 log (cfu/g) [54]. Beef meat sold at the street of Jijiga, Somali region of Ethiopia, was higher (8.07 log (cfu/g)) [55] than this study (7.85 log (cfu/g)). Fairly similar result was detected in mesophilic count (6.68 cfu) in a study conducted in raw beef meat in Addis Ababa [56]; the total coliform was 6.4 versus 6.5 in the present study and fecal coliform 5.82 versus 6.73 log (cfu/g). However, mold and yeast counts were higher in the above studies (2.25 versus 5.82 log (cfu/g)).

A study conducted in Adama, Ethiopia, reported that lower aerobic mesophilic count (5.20) detected total coliform (1.72) and fecal coliform (1.96), but higher amount of E. coli (1.95 log (cfu/g)) was detected in raw beef meat compared to the current study [57]. A study in ground beef meat in Sudan examined lower aerobic mesophilic count (0.76) and E. coli (0.77 log (cfu/g)) than the present study [58]. There are limited published articles on microbial content of meat powder. The nutritional composition of dry meat powder is concentrated when it is in dry form, thus able to satisfy a variety of macro- and micronutrient needs of infants and young children. If a child uses one tablespoon of it in his/her complementary food three times a day, it can fulfill 70% of energy need, seven times the protein requirement, 50% of daily fat, 70% of phosphorus, 1/3 of daily zinc, and 10% iron requirement have been covered. Most of the nutrients in dry meat powder are significantly higher compared to raw meat because the water constituent of raw meat is very high, which becomes massively concentrated as reported by [15].

## 5. Limitation of the Study

The study explored major nutrient profile of dried meat powder and its safety, conducted animal trial to see the toxicity of it, and examined organ histopathology such as liver and kidney, coupled with analyzing organ function tests. However, small intestine histopathology sectioning or microscopical examination should have been conducted which would indicate whether presence or absence of problems is associated with gastrointestinal tract.

## 6. Conclusion

Meat, especially in the form of meat powder, is loaded with macro- and micronutrients which could support growth and fulfill nutritional needs of children among 6-23 months old via sprinkling in their complementary foods. Feeding of meat powder and its mixture for rats revealed absence of any liver or kidney histopathology, nor indicated exaggerated results of organ function tests in 30 days of feeding period. Since meat is one of the favorable foods for bacterial proliferation during production, packaging, distribution, and storage, thus cautions are compulsory to avoid contamination and keeping its hygiene in the value chain. Optimal nutrient composition and absence of histopathological findings in the present study stimulate a follow-up study of human clinical trial. Moreover, further investigation is recommended on the nutrient content and sensory acceptability of dried beef meat powder mixed with mainly used children's complementary foods.

## Figures and Tables

**Table 1:** Nutrient composition of standard rat diet as labeled in product package.

Nutrient type	Amount (%)	Nutrient type	Amount (g/kg)
Crude protein	16.00	Phosphorus	5.50
Crude fat	3.10	Potassium	6.00
Crude ash	4.60	Sodium	2.50
Crude fiber	3.90	Calcium	7.30
Moisture	11.90	Iron	0.27
Utilizable carbohydrate	60.40	Zinc	0.06
Energy (kcal/100 g)	333.90		

**Table 2:** Weight gain, food intake, and food efficiency ratio of rats fed with standard, mixed, and dry meat.

Parameters	Standard diet-fed rats (*n* = 12)	Mixed diet-fed rats (*n* = 12)	Dry meat-fed rats (*n* = 12)
Mean initial wt (g)	129.20 ± 35.85	129.17 ± 35.86	125.83 ± 37.71
Mean final wt (g)	239.58 ± 34.08	239.58 ± 34.08	210.42 ± 21.47
Weight gain (g)	77.92 ± 8.97^a^	110.42 ± 10.65^a^	84.58 ± 10.83^a^
Mean food intake (g/day)	18.37 ± 0.55^a^	17.57 ± 0.47^a^	13.28 ± 0.57^b^
Food efficiency ratio (%)	4.16 ± 0.42^b^	6.23 ± 0.50^a^	6.22 ± 0.72^a^

Group I = standard diet; group II = standard diet+dry meat powder (50 : 50%); group III = dry meat powder only. The results are presented in mean ± SEM. Means presented with the same letters across a row indicate no significant difference at *p* < 0.05.

**Table 3:** Liver, kidney, and heart function tests of rats feed with a standard diet, mixed, and dry meat powder.

Parameters	Standard (*n* = 12)	Mixed (*n* = 12)	Meat powder (*n* = 12)
ALT (U/L)	24.43 ± 3.14^b^	23.27 ± 2.23^b^	40.31 ± 5.19^a^
AST (U/L)	118.03 ± 11.74	127.92 ± 12.43	122.67 ± 13.81
ALP (U/L)	81.83 ± 13.79^b^	77.83 ± 4.97^b^	200.08 ± 26.42^a^
ALB (g/dL)	3.49 ± 0.31	3.72 ± 0.13	3.61 ± 0.29
BILT (mg/dL)	0.03 ± 0.00^ab^	0.02 ± 0.00^b^	0.05 ± 0.00^a^
AMYL (U/L)	1552.58 ± 156.45	1478.92 ± 59.08	1521.54 ± 138.01
LIP (U/L)	3.08 ± 0.42	2.58 ± 0.11	2.87 ± 0.21
CRP-HS (mg/L)	0.62 ± 0.10	0.40 ± 0.09	0.44 ± 0.10
Troponin (pg/mL)	4.91 ± 0.44	5.20 ± 0.17	5.36 ± 0.42
CK (U/L)	410.25 ± 52.49^b^	530.90 ± 43.66^ab^	638.33 ± 84.82^a^
Urea (mg/dL)	37.03 ± 3.18^b^	41.71 ± 2.13^b^	75.98 ± 3.11^a^
Creatinine (mg/dL)	0.29 ± 0.02	0.30 ± 0.01	0.32 ± 0.03

Results are presented in mean ± SEM; means of the same letters across a row indicate no significant difference at *p* < 0.05. Mix = standard diet+dry meat powder; ALT = alanine aminotransferase; AST = aspartate aminotransferase; ALP = alkaline phosphatase; ALB = albumin; BILT = bilirubin total; AMYL = *α*-amylase; LIP = lipase; CRP-HS = highly sensitive C-reactive protein; CK = creatine kinase.

**Table 4:** Beef meat powder nutrient composition from samples collected in different weeks.

Parameter	Week I	Week II	Week III	Average
Moisture (g/100 g)	7.61 ± 0.09	8.75 ± 0.26	8.27 ± 0.01	8.21 ± 0.54
Total ash (g/100 g)	7.28 ± 0.17	4.95 ± 0.45	7.12 ± 0.03	6.45 ± 1.21
Crude protein (g/100 g)	71.75 ± 0.64	76.85 ± 0.72	79.76 ± 0.28	76.12 ± 3.68
Crude fat (g/100 g)	10.7 ± 0.48	7.36 ± 0.33	6.51 ± 0.01	8.19 ± 2.01
Crude fiber (g/100 g)	0.37 ± 0.14	0.28 ± 0.05	1.03 ± 0.03	0.56 ± 0.38
Utilizable carbohydrate (g/100 g)	2.30 ± 0.04	3.02 ± 0.05	3.05 ± 0.07	2.79 ± 0.38
Phosphorus (mg/100 g)	138.47 ± 2.77	142.58 ± 7.03	170.02 ± 3.94	150.35 ± 16.26
Potassium (mg/100 g)	858.97 ± 28.25	721.38 ± 4.00	718.14 ± 56.42	766.16 ± 77.26
Sodium (mg/100 g)	99.86 ± 3.99	105.78 ± 2.0	165.67 ± 1.99	123.76 ± 32.71
Calcium (mg/100 g)	8.80 ± 1.46	25.75 ± 0.93	19.91 ± 1.45	18.15 ± 7.80
Zinc (mg/100 g)	3.90 ± 0.09	3.76 ± 0.05	3.80 ± 0.10	3.82 ± 0.10
Iron (mg/100 g)	2.81 ± 0.08	2.76 ± .08	2.83 ± 0.15	2.80 ± 0.09
Energy (kcal/100 g)	392.46 ± 1.52	385.66 ± 0.14	389.79 ± 1.06	389.30 ± 3.25

**Table 5:** Commutative mean of microbial log (cfu/g) for standard, mixed, and dry red meat powder.

Microorganism	Standard diet	Mix diet	Meat powder
	Mean ± SEM	Mean ± SEM	Mean ± SEM
Aerobic mesophilic	4.74 ± 0.31	5.53 ± 0.46	7.85 ± 0.12
Total coliform	3.14 ± 0.38	4.11 ± 0.31	6.47 ± 0.61
Fecal coliform	1.43 ± 0.1	3.46 ± 0.04	6.2 ± 0.33
E. coli	1.43 ± 0.1	3.49 ± 0.27	3.96 ± 0.8
Mold	2.71 ± 0.38	2.64 ± 0.48	2.25 ±0.71
Yeast	ND	ND	ND
Salmonella per 25 g	Absent	Absent	Absent

ND = not detected.

**Table 6:** Daily nutrient requirements and amount fulfilled in the ration.

Energy and nutrients in present study	Average value (100 g)	Amount in 3 × 14 g	Nutrients require per daily ration for 6-23 months	Requirement fulfilled (%)
Energy (kcal)	389.30	163.51	220.00	74.32
Protein (g)	76.12	31.97	3.00–5.50	750.59
Fat (g)	8.19	3.44	6.30	54.60
Phosphorus (mg)	150.36	63.15	75.00–100.00	72.17
Potassium (mg)	766.16	321.79	NA	NA
Calcium (mg)	18.15	7.62	100.00–200.00	5.08
Zinc (mg)	3.82	1.60	4.00–5.00	35.65
Iron (mg)	2.80	1.18	7.00–11.00	13.07

Two tablespoon of meat powder ~3^∗^14 g = 42 g. NA = not applicable.

## Data Availability

Data will be available based on request to corresponding author.

## Abbreviations

ALB: Albumin
ALP: Alkaline phosphatase
ALT: Alanine transaminase
AMYL: *α*-Amylase
AOAC: Association of Official Analytical Chemists
AST: Aspartate transaminase
BILT: Bilirubin total
CFU: Colony-forming units
CRP-HS: C-reactive protein high sensitive
CK: Creatine kinase
LIP: Lipase
EPHI: Ethiopian public health institution
PUFAs: Polyunsaturated fatty acids.
